# From monocots to dicots: the multifold aspect of cell wall expansion

**DOI:** 10.1093/jxb/eraa573

**Published:** 2021-02-27

**Authors:** Kalina T Haas, Alexis Peaucelle

**Affiliations:** Institut Jean-Pierre Bourgin, INRAE, AgroParisTech, Université Paris-Saclay, Versailles, France

**Keywords:** biomechanics, cell wall, endocytosis, growth, morphogenesis, pectins

## Abstract

This article comments on:

**Petrova AA, Gorshkova TA, Kozlova LV**. 2021. Gradients of cell wall nano-mechanical properties along and across elongating primary roots of maize. Journal of Experimental Botany **72**, 1764–1781.

**The survival of our civilization is entirely dependent on plants. Nevertheless, many relevant aspects of plant biology remain unknown. One of the most intriguing is the biological process of plant growth and morphogenesis. The conundrum of plant growth lies in the rigid polysaccharide network surrounding every plant cell: the cell wall (** Caffall and Mohnen, 2009 **). It determines cellular shapes, tissue biomechanics, and the overall morphology of the organism (** Höfte and Voxeur, 2017 **). Ultimately the growth process in plants depends on the cell wall expansion allowing cell enlargement.**

Direct exploration of the cell wall structure revealed that its polymers are distributed anisotropically: cellulose is deposited in the cell wall in parallel fibrils perpendicular to the growth axis (Gutierrez *et al.*, 2009; Anderson *et al.*, 2010). This is achieved by the cellulose synthase complex residing in the membrane moving parallel to microtubules (Bringmann *et al.*, 2012). The perturbation in cellulose synthase or microtubule organization does not inhibit growth but annihilates its asymmetry, leading to isotropic tissue expansion (Peaucelle *et al.*, 2015). These experiments reveal that cellulose organization restricts the growth locally, allowing anisotropic expansion. It was postulated that growth is accomplished by loosening the cellulose network (Cosgrove, 2016). The observation that xyloglucan interacts with cellulose microfibrils at their junctions suggested that cell wall loosening could depend on xyloglucan remodeling. Atomic force microscopy (AFM) associated with creep experiments revealed that tension-induced cell wall expansion is associated with cellulose fiber reorientation, parallel to the expansion, facilitated by xyloglucan remodeling (Zhang *et al.*, 2017). However, the null mutant in xyloglucan in Arabidopsis does not present severe growth defects (Park and Cosgrove, 2012). Thus, xyloglucan is implicated in the growth process, but not as the driving element.

A different approach to understanding the growth process concentrates on the hormonal and genetic regulation of morphogenesis. This research permitted determination of the elements controlling differential growth and cell differentiation programs (Vanstraelen and Benkov, 2012). The focus on the role of the phytohormone auxin in organ formation in the apical meristem revealed other aspects of cell wall expansion (Reinhardt *et al.*, 2000). First, it indicated that auxin’s local accumulation is sufficient and necessary to induce lateral organ formation. Secondly, it is associated with pectin chemistry changes directly in the cell wall—notably, auxin accumulation-induced de-methylesterification of the pectin homogalacturonan (HG). Furthermore, HG de-methylesterification was also demonstrated to be necessary and sufficient to lead to cell expansion at the position of the primordia and that it is required for auxin primordia induction (Braybrook and Peaucelle, 2013; Peaucelle et al., 2011*a*, *b*). These observations infer a strong correlation between pectin chemistry changes and cell growth. The pioneering work on pollen tube growth has revealed that pectin methylation changes are correlated with alterations in cell wall elastic properties (Chebli *et al.*, 2012). The correlation between cell wall elasticity changes and growth was confirmed in several tissues (Peaucelle *et al.*, 2015; Peaucelle *et al.*, 2011*a*; Majda *et al.*, 2017; Feng *et al.*, 2018; Schoenaers *et al.*, 2018; Li *et al.*, 2020, Preprint); see also references in Petrova *et al.*(2021). Such drastic importance for a simple chemical change of the polymer considered until then as merely a cell wall padding is complicated to understand. Moreover, monocotyledons have significantly reduced pectin content in their cell walls; does this imply that monocots evolved a distinct cell wall growth mechanism, or are HG-related growth mechanisms an epiphenomenon? The HG effect on growth through an increase in cell wall elasticity is counter-intuitive. First, demethylated pectins dimerize through calcium ion bridges, which jellifies the HG network and, supposedly, augments wall rigidity. Secondly, in mechanical terms, growth is an irreversible deformation of the cell wall, whereas elasticity is reversible. How could elasticity be linked to growth?

The proper way to bring back clarity in plant growth studies was to explore the chemical and mechanical events linked to monocotyledons’ growth. In this issue of the journa, Petrova and colleagues (2021) used the AFM approach to measure the cell wall’s mechanical properties in the root apex of maize. This work gives one of the most detailed mechanical maps of a full organ described in plant tissue. The observed mechanical properties were introduced in a mechanical model to predict stress distribution. The mechanical measurements were complemented with semi-quantitative cartography of the cell wall polymer using immunolabeling. Nevertheless, comparison of the mechanical and chemical maps did not reveal a direct correlation between cell wall polymer distribution and the cell wall’s rheological characteristics. It is deceptive, but it was predictable; cell wall components are often redundant in their function, as suggested for the xyloglucans’ null mutant. However, an increase in demethylated HG in the elongating zone compared with the meristem correlated with growth and elasticity, similarly to what was observed in Arabidopsis organs (Peaucelle *et al.*, 2015). A decrease in the HG methylation during the elongation stage was previously observed in maize roots (Kozlova *et al.*, 2020). This hints that even in monocotyledons, HG demethylation plays an essential role in plant growth. This work is a landmark in understanding the cell wall chemistry and its relationship to growth and mechanics in monocotyledons. This study paved the way for future research to prioritize exploring the cell wall polymer architecture and organization in three dimensions and link it to the biomechanical characteristics. Biomechanical properties and sensing are essential to shaping the macroscale phenotypes (Fruleux *et al.*, 2019). To advance our understanding of growth, it is essential to create an atlas of cell wall organization and chemistry based on reliable techniques. With this map, we would finally be able to navigate the structure–function relationship of the different cell wall polymers. The cell wall ultrastructure is often observed using AFM and EM; however, both techniques lack biochemical contrast, and inferring the polymer identity is not straightforward. Immunogold (i)EM compensates for the lack of molecular specificity (Majda *et al.*, 2017); yet the plastic embedding leads to a low density of recognized epitopes and reduced effective resolution. Recently, single-molecule nanoscopy empowered the semi-quantitative high-density mapping of the cell wall polymer organization in 3D and with a resolution of 50 nm (Haas *et al.*, 2020). It revealed an unexpected structural element of the cell wall, HG nanofilaments, that weakened previous cell wall structure and function assumptions. Seemingly unforeseen, these observations are supported by the numerous observation made since the 1930s (van Iterson, 1933; Palmer *et al.*, 1947; Roelofsen and Kreger, 1951; Sterling, 1957; Leppard and Colvin, 1971; Ramamoorthy and Leppard, 1977; Arend *et al.*, 2008). The thread-like pectin with a fine structure composed of axially aligned HG polymers arranged on a crystal grid was reported *in vitro* and in various plant tissues. These observations inspired an alternative vision of plant cell growth where pectin demethylation leads to cell wall expansion without the turgor pressure, underpinned by the rearranging of HG polymers on a different lattice, from the compact hexagonal to a looser rectangular net (Haas *et al.*, 2020). This model explains the paradoxical correlation between the growth, cell wall elasticity, and pectin demethylation. The alteration in the cell wall elasticity would be an epiphenomenon of the polymer density change due to swelling, water affinity, and the conformation of cell wall polymers. This paradigm change indicates new parameters such as HG secretion, HG nanofilament organization, and spatiotemporal change in the HG demethylation pattern.

Plant growth is highly dynamic, characterized by waves of growth observed at the single-cell level on pollen tubes or root hairs, and at the whole-organ level as plant’s growth motions (nutation) (Schoenaers *et al.*, 2018). Thus, growth is fundamentally an oscillatory process that needs to be explored with a resolution of minutes, if not seconds. Pectin demethylation is one of the crucial modifications that require dynamic observations with high spatiotemporal precision. The cell plate assembly is one example of the cell wall chemistry dynamics that includes polymer degradation (Dhonukshe *et al.*, 2006; Peaucelle *et al.*, 2020). Endocytosis of xyloglucan, rhamnogalacturonan, and HG was already demonstrated (Baluška et al., 2002, 2005*a*, *b*). We conjecture that cell wall polymer recycling could be more prevalent than generally thought. The immunohistochemistry approach only gives a steady-state quantification. A full account of the cell wall chemistry related to growth would need to include the dynamic range of the synthesis, *in muro* chemical reorganization, degradation, and recycling of different polysaccharides. We speculate that the recycling of the HG after demethylation is an essential aspect of cell expansion. We envisage that recycling is boosted in monocotyledons, explaining the reduced amount of HG in their primary cell walls. The previously observed transient increase in xyloglucans and HG synthesis at early elongation (Kozlova *et al.*, 2020) and a lowered HG level in older roots observed in Petrova *et al*. (2021) support this hypothesis. This growth characteristic could be fundamental to the monocotyledon evolution, rapid annual growth, and selection as crops during the Neolithic. The combined effort integrating biomechanics, modeling, polymer chemistry, genetics, and next-generation nanoimaging should soon uncover processes at the heart of plant growth.
